# Translation Initiation Factor eIF4E and eIFiso4E Are Both Required for *Peanut stripe virus* Infection in Peanut (*Arachis hypogaea* L.)

**DOI:** 10.3389/fmicb.2017.00338

**Published:** 2017-03-10

**Authors:** Manlin Xu, Hongfeng Xie, Juxiang Wu, Lianhui Xie, Jinguang Yang, Yucheng Chi

**Affiliations:** ^1^Shandong Peanut Research InstituteQingdao, China; ^2^Fujian Agriculture and Forestry UniversityFuzhou, China; ^3^Open Project Program of Key Laboratory of Tobacco Pest Monitoring Controlling and Integrated Management, Tobacco Research Institute of Chinese Academy of Agricultural SciencesQingdao, China

**Keywords:** peanut, *Peanut stripe virus*, translation initiation factor 4E, protein–protein interaction, gene silencing

## Abstract

*Peanut stripe virus* (PStV) belongs to the genus *Potyvirus* and is the most important viral pathogen of cultivated peanut (*Arachis hypogaea* L.). The eukaryotic translation initiation factor, eIF4E, and its isoform, eIF(iso)4E, play key roles during virus infection in plants, particularly *Potyvirus*. In the present study, we cloned the *eIF4E* and *eIF(iso)4E* homologs in peanut and named these as *PeaeIF4E* and *PeaeIF(iso)4E*, respectively. Quantitative real-time PCR (qRT-PCR) analysis showed that these two genes were expressed during all growth periods and in all peanut organs, but were especially abundant in young leaves and roots. These also had similar expression levels. Yeast two-hybrid analysis showed that PStV multifunctional helper component proteinase (HC-Pro) and viral protein genome-linked (VPg) both interacted with PeaeIF4E and PeaeIF(iso)4E. Bimolecular fluorescence complementation assay showed that there was an interaction between HC-Pro and PeaeIF4E/PeaeIF(iso)4E in the cytoplasm and between VPg and PeaeIF4E/PeaeIF(iso)4E in the nucleus. Silencing either *PeaeIF4E* or *PeaeIF(iso)4E* using a virus-induced gene silencing system did not significantly affect PStV accumulation. However, silencing both *PeaeIF4E* and *PeaeIF(iso)4E* genes significantly weakened PStV accumulation. The findings of the present study suggest that PeaeIF4E and PeaeIF(iso)4E play important roles in the PStV infection cycle and may potentially contribute to PStV resistance.

## Introduction

Peanut is one of the most important oil crops and food legumes in the world. In China, peanuts are grown on 3.5 million hectares of land each year (Xu, 2008). *Peanut stripe virus* (PStV; genus *Potyvirus*, family *Potyviridae*) is one of the most widely distributed peanut viruses constraining peanut production. PStV has been detected in various countries, including China, the US, the Philippines, Thailand, Indonesia, Malaysia, and Korea (Xu et al., 1983; Demski and Lovell, 1985; Saleh et al., 1989; Choi et al., 2001, 2006). Recently, PStV has been reported in India and some African countries, which were possibly caused by exchanges in peanut seed resources (Xu, 2008).

In China, PStV is a very serious infectious disease that afflicts peanut, particularly those grown in northern China. The infection incidence has reached 50% and recently, even 100% in some fields. PStV has also infected various other crops, including soybean (*Glycine max*), sesame (*Sesamum indicum*), cowpea (*Vigna unguiculata*), hyacinth bean (*Dolichos lablab*), white lupin (*Lupinus albus*), and patchouli (*Pogostemon cablin*; Xu, 2008; Singh et al., 2009). To date, no effective method for controlling this virus has been established.

*Peanut stripe virus* is a member of the genus *Potyvirus*, an economically significant and one of the largest groups of viruses that infect plants. These viruses are about 10 kb in length, carry a single positive-strand RNA, and contain a 350-kD polyprotein that is translated by a single open reading frame (ORF). The polyprotein is cleaved by three virus-encoded proteases into 10 mature proteins and an additional protein called PIPO, which is embedded in the P3 cistron (Urcuqui-Inchima et al., 2001; Wei et al., 2010).

One of the three virus-encoded proteases is the multifunctional helper component proteinase (HC-Pro), which consists of C-proximal, central, and N-proximal domains. The C-proximal domain separates HC-Pro from the polyprotein precursor via proteolysis (Carrington and Herndon, 1992). HC-Pro contributes to various essential steps that are related to viral replication and infection cycles. HC-Pro is involved in some processes, including virus transmission by aphids (Govier et al., 1977) and virus movement from cell-to-cell (Rojas et al., 1997) to long-distance migration (Saenz et al., 2002). In addition, HC-Pro facilitates the development of virulence and symptom amplification (Atreya et al., 1992; Redondo et al., 2001); it is also a regulator of gene silencing suppression (Llave et al., 2000). HC-Pro interacts with numerous host proteins and some virus proteins, as well as mediates the function of host proteins and other viral proteins (Jin et al., 2007; Ala-Poikela et al., 2011).

Another important region of the potyviral protein is VPg, which is translated into the polyprotein, NIa, which is also known as VPg-Pro. During the potyvirus infection, VPg participates in replication and proteolysis and is composed of N-terminal and C-terminal protease domains (Revers et al., 1999). VPg is a multifunctional protein that plays a crucial role in race-specific replication (translation and RNA synthesis), as well as in cell-to-cell and long-distance movement; it also interacts with host proteins as well as various recessive potyvirus resistance genes in different host species (Lellis et al., 2002; Rajamaki and Valkonen, 2002).

Because viruses have relatively small genomes and a limited number of proteins, they rely on the host-cell environment to complete their infection cycle. The characterization of host proteins, membranes, and nucleic acids, using a model host system, functional genomics, and modern molecular biology methods, help in the understanding of plant–virus interactions (Whitham and Wang, 2004). For example, positive-sense ssRNA viruses replicate in association with host endomembranes (Mackenzie, 2005) and different host factors (Ahlquist et al., 2003; Whitham and Wang, 2004). One of the most important genes is the translation initiation factor, *eIF4E*, which initiates the translation of mRNA and regulates protein synthesis (Sonenberg et al., 1978; Jackson et al., 2010). *eIF4E* also interacts with the 5′-terminal cap of mRNA and was initially named the ‘cap-binding protein.’ Moreover, *eIF4E* and its isoform *eIF(iso)4E* are functionally redundant and one or both of them interact with HCpro and VPg, which are indispensable for viruses to complete their infection cycle; therefore, abolishing this interaction may prevent the viral infection (Lellis et al., 2002; Browning, 2004; Kang et al., 2005; Jin et al., 2007; Charron et al., 2008; Ala-Poikela et al., 2011; Wang and Krishnaswamy, 2012; Sanfacon, 2015). Based on this concept, silencing or incurring mutation in the gene may disrupt infection. *pvr2* is a two-nucleotide substitution of the amino acid of pepper eIF4E and is resistant to PVY (Ruffel et al., 2002). A small number of amino acid substitutions in the tomato eIF4E *pot-1* confer resistance against PVY and *Tobacco etch virus* (TEV) in tomato. Barley *rym4* and *rym5* are also amino acid substitutions that confer eIF4E resistance to *Barley yellow mosaic virus* (BaYMV) and *Barley mild mosaic virus* (BaMMV) in Barley (Kanyuka et al., 2005; Stein et al., 2005). Pepper *pvr1*(*2*) contains an eIF4E mutation and *pvr6* is an eIF(iso)4E mutation; simultaneous mutations in eIF4E and eIF(iso)4E confer resistance to *Chilli veinal mottle virus* (ChiVMV) in pepper, and silencing eIF4E and eIF(iso)4E reduces the ChiVMV accumulation (Ruffel et al., 2006; Hwang et al., 2009). In plum, the silencing of eIF(iso)4E results in resistance to *Plum pox virus* (PPV; Wang et al., 2013; Cui and Wang, 2016). Thus, the dependence of potyviruses on eIF4E and/or eIF(iso)4E varies with each virus–host interaction.

To date, no effective way of controlling PStV such as using genetically resistant varieties has been established, mainly because no resistance genes have been identified. We hypothesize that peanut eIF4E/eIF(iso)4E controls the effect of PStV in peanut. To test this hypothesis, we investigated the effects of silencing the translation initiation factor, eIF4E/eIF(iso)4E, to confer PStV resistance in peanut. Moreover, we examined the interaction between HC-Pro and VPg of PStV with eIF4E/eIF(iso)4E using Y2H and BiFC. We also detected the expression of eIF4E/eIF(iso)4E in different peanut tissues.

## Materials and Methods

### Cloning and Sequencing of *PeaeIF4E* and *PeaeIF(iso)4E* Genes

Total RNA was extracted from peanut (*Arachis hypogaea*) leaves using TRIzol (Invitrogen, Carlsbad, CA, USA), and cDNA was synthesized using an M-MLV RTase cDNA synthesis kit (Takara, Dalian, China), following the manufacturer’s recommendations. To design primers for cloning the *eIF4E* and *eIF(iso)4E* genes of peanut, we compared and downloaded the *eIF4E* sequences of *Medicago truncatula* (XM_003593785), *Pisum sativum* (AY423375), *Pisum sativum* (DQ641471), *Phaseolus vulgaris* (EF571276), *Phaseolus vulgaris* (EF571275), *M. tornata* (HQ735878), and *M. truncatula* (HQ735877). The conserved sequences were used in designing the primer pairs PeaeIF4E2-R and PeaeIF4E2-F (Supplementary Table S1) to amplify the peanut *eIF4E* gene. The PCR products showing the expected lengths were sequenced and compared. The product with the correct sequence was then used in designing primers (Supplementary Table S1) for 5′ amplification of cDNA ends (5′-RACE) and 3′-RACE to obtain the full-length cDNA of *PeaeIF4E*. A 5′-RACE kit (Invitrogen) was used according to the manufacturer’s instructions to obtain the 5′ terminus of the *PeaeIF4E* gene. The 5′-RACE *eIF4E* outer and inner primers and 3′-RACE *eIF4E* outer and inner primer (Supplementary Table S1) were used to obtain the full-length *PeaeIF4E* cDNA. The *PeaeIF(iso)4E* gene was amplified using the primers listed in Supplementary Table S1. Phusion high-fidelity DNA polymerase (Takara, Dalian, China) was used to perform all the PCRs. A gel extraction kit (TianGen, Beijing, China) was used to purify the PCR products, which were then cloned into a pMD-18T easy vector (Takara) for sequencing. DNAMAN 6.0 was used for multiple sequence alignment to homologous proteins of different plant species. MEGA5 with the Equal input model was used for phylogenetic analyses by using the neighbor-joining (NJ) method, and confidence was estimated by using 1,000 bootstrap replicates (Tamura et al., 2011).

### Cloning and Sequencing of PStV VPg and HC-Pro

Total RNA extraction and cDNA synthesis of the PStV VPg and HC-Pro genes were similar to the method used in cloning *PeaeIF4E* and *PeaeIF(iso)4E*. Based on the reported cDNA sequence of PStV (GenBank Accession No.: KF439722, U05771, and U34972), we designed primers for the amplification of segments that corresponded to PStV VPg and PStV HC-Pro (Supplementary Table S1). Phusion high-fidelity DNA polymerase (Takara) was used for all PCRs. A gel extraction kit (TianGen) was used to purify the PCR products. Then the purified PCR products were cloned into a pMD-18T easy vector (Takara) for sequencing.

### qRT-PCR Analysis

Total RNA samples were extracted from the roots, stems, leaves, flower buds, leaf buds of “Huayu 20,” and cDNAs were synthesized using the same method employed in cloning the *PeaeIF4E* and *PeaeIF(iso)4E* genes. All peanut tissues were sampled from three different peanut plants as biological replicates. For the analysis of gene silencing in peanut, total RNA was extracted from the leaves of eIF4E-silenced, eIF(iso)4E-silenced, or eIF4E-eIF(iso)4E-double silenced peanuts and used in RT-PCR as previously described. Quantitative real-time PCR (qRT-PCR) reactions were conducted by using a SYBR *Premix Ex Taq* PCR kit (Takara) on an ABI7500 real-time PCR system (ABI, Foster, CA, USA). The primer pairs YG4E-R/YG4E-F and YG4IE-R/YG4IE-F were used to detect the expression of *PeaeIF4E* and *PeaeIF(iso)4E*. The primer pair YGPStV-R/YGPStV-F was used to detect the accumulation of PStV after inoculation. The primer pair actin-R/actin-F was used to amplify the *actin* gene of *A. hypogaea*, which was used as a reference. The PCR reaction system consisted of a total volume of 20 μL, which included 2 μL of the RT product, 10 μL of Ex Taq, 0.8 μL of the primers (Supplementary Table S1), and 7.2 μL of DEPC-water. All the reactions were performed in a 96-well optical plate. The PCR conditions were as follows: 94°C for 15 s, 94°C for 6 s, and 60°C for 30 s for a total of 40 cycles. Data analysis was performed by using an ABI7500 real-time PCR system, and standard curves were also constructed.

### Subcellular Localization of PStV VPg, PStV HC-Pro, and eIF4E/eIF(iso)4E

The ORF of the target gene without its stop codon was amplified using the Phusion high-fidelity DNA polymerase (Takara) using the corresponding primer pairs, ORF4E-R/ORF4E-F, 4E(isoORF)F/4E(isoORF)R, PV-F/PV-R, and PH-R/PH-F, and then cloned into a pMD-18T easy vector (Takara) for sequencing. After confirmation of the correct clone from pMD-18T, these were then introduced into an entry vector pGWCm by TA cloning, and finally, via LR gateway recombination reaction (Invitrogen), was transferred to the plant expression vector, pHZM03. Plasmid DNA with green fluorescent protein (GFP) was transiently introduced into *Arabidopsis* protoplasts (Meng, 2012). After incubating for 12–16 h in the dark, GFP expression was visualized using a confocal laser microscope (Leica SP5, Mannheim, Germany).

### Yeast Two-Hybrid Assay

Yeast two-hybrid screening was conducted using a Matchmaker Gold Yeast two-hybrid system (Clontech, Mountain View, CA, USA). The coding sequences of PStV VPg, PStV HC-Pro, and eIF4E/eIF(iso)4E were PCR amplified by using Phusion high-fidelity DNA polymerase (Takara) with the primer pair listed in Supplementary Table S1. PStV VPg, PStV HC-Pro were cloned into the prey vector, pGADT7, and eIF4E/eIF(iso)4E were cloned into the bait vector, pGBKT7. Confirmed correct clones were transformed into *Escherichia coli* DH5α cells for subsequent DNA sequencing. Both the confirmed correct prey and bait vectors were then co-transformed into AH109 yeast cells. SD/-Leu-Trp, SD/-Leu-Trp-His, SD/-Leu-Trp-His-Ade, and SD/-Leu-Trp-His-Ade+X-α-gal (Clontech) were used as selective media to detect any interactions. Yeast that contained both empty pGADT7 and pGBKT7 were used as negative controls, and yeast containing both pGBK-p53 and pGAD-RecT were used as positive controls.

### Bimolecular Fluorescence Complementation (BiFC)

The Gateway compatible BiFC vectors pEarleyGate202-NYFP and pEarleyGate202-CYFP were used. DNA fragments corresponding to PStV VPg, PStV HC-Pro, and eIF4E/eIF(iso)4E were introduced individually into the entry vector pGWCm as previously described. pGWCm-VPg and pGWCm-HC-pro were transferred to the pEarleyGate202-CYFP vector, whereas pGWCm-eIF4E and pGWCm-eIF(iso)4E were transferred to the pEarleyGate202-NYFP vector via the LR Gateway recombination reaction (Invitrogen). Plasmid DNA with YFP fusion was introduced into *Arabidopsis* protoplasts for transient expression (Meng, 2012). After incubation for 12–16 h in the dark, confocal laser-scanning microscopy (Leica SP5) was performed to evaluate YFP expression.

### Silencing of Peanut Using the Virus-Induced Gene Silencing (VIGS) Vector System

For VIGS assays, *PeaeIF4E* and *PeaeIF(iso)4E* were amplified by using the primers listed in Supplementary Table S1. The PCR products were digested with *Xho*I and *BamH*I (New England Biolabs) and ligated into vector ALSV-RNA2, which was digested with the corresponding enzymes. The plasmid constructs were then sequenced (Takara) to confirm that we obtained the correct inserts. *Apple latent spherical virus* (ALSV)-based VIGS method was used. The constructed ALSV-RNA2 and ALSV-RNA1 were transformed into *E. coli* DH5α cells for sequencing; then the confirmed correct plasmid was cultured in *E. coli* DH5α cells using TransGEN plasmid Maxi Kit (TransGEN) for purification. Approximately 1 μg/μL of modified ALSV-RNA2 and ALSV-RNA1 were mechanically inoculated into *Chenopodium quinoa* plants. Two to three weeks later, symptomatic leaves were collected, homogenized in three volumes of extraction buffer (0.1 M Tris-HCl, pH7.8, 0.1 M NaCl, 5 mM MgCl_2_), and re-inoculated in *C. quinoa* plants. Then the total RNA of symptomatic leaves were extracted and diluted to about 0.02 μg/μL to be mechanically inoculated into peanuts (2 weeks after sprouting; Igarashi et al., 2009).

### Virus Inoculation

In the present study, the PStV isolate was from Laixi, Qingdao city, Shandong province, China. It was cultured in our laboratory via mechanical inoculation for maintenance. PStV-infected plants were maintained at 25°C with 8 h photoperiod. Two weeks after silenced peanut, PStV was mechanically inoculated into peanut. After infection, the presence of PStV was tested by real-time RT-PCR.

## Results

### Cloning and Sequencing of *PeaeIF4E* and *PeaeIF(iso)4E* Genes

The sequence of the peanut *eIF4E* gene was amplified by using 5′-RACE and 3′-RACE. The full-length cDNA sequence of the *eIF4E* gene was deposited in GenBank (Accession No. HE985069). The *eIF4E* gene was 764-bp long, with a 5′ 39-bp untranslated region, a 696-bp ORF, and a 29-bp 3′ untranslated region, and encoded a putative 231-amino acid polypeptide. Moreover, it had 92% nucleotide sequence identity to its homolog in *Pisum sativum cultivar* (GenBank Accession No. AY423375.2) and 70% nucleotide identity to its homolog in *M. truncatula* (GenBank Accession No. BT134162.1).

The peanut *eIF(iso)4E* gene was isolated as described above. The obtained peanut *eIF(iso)4E* ORF was 612 bp (GenBank Accession No. KF956378) and was predicted to encode a 203-amino acid polypeptide. The cloned peanut *eIF4E* and *eIF(iso)4E* genes were designated as *peaeIF4E* and *peaeIF(iso)4E*, respectively. The identity of the *peaeIF4E* and *peaeIF(iso)4E* genes at the nucleotide sequence level was 53.55%, whereas that at the amino acid sequence level was 42.67%.

**FIGURE 1:**
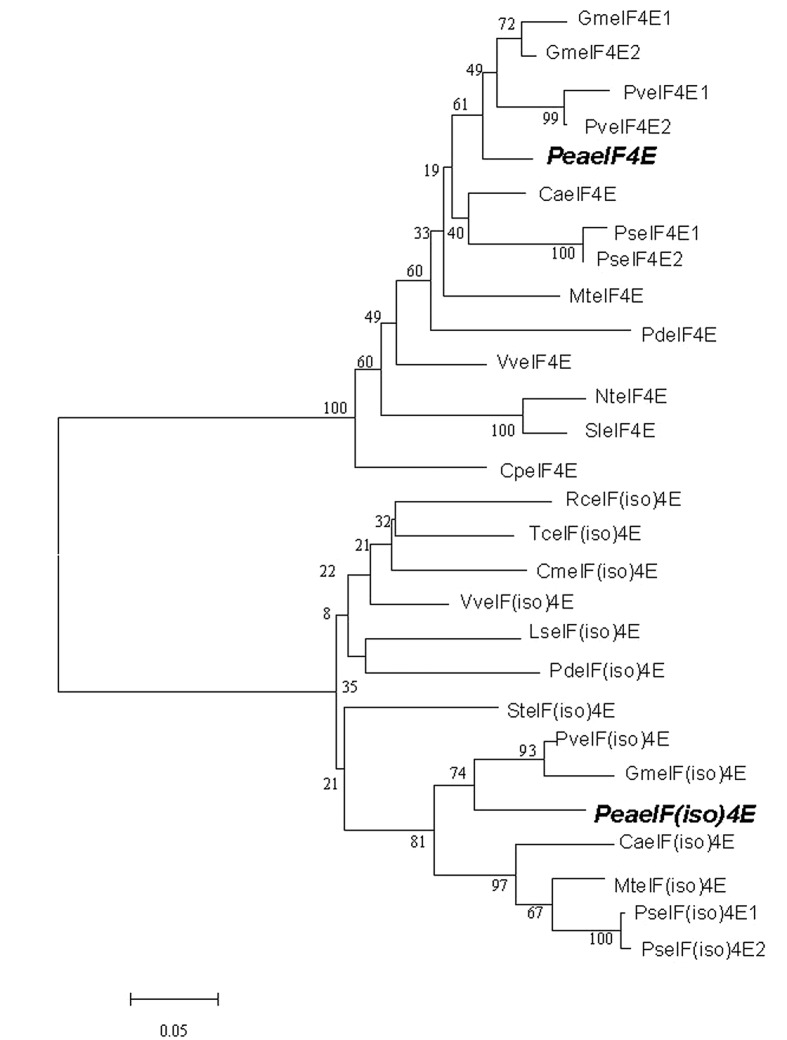
**Phylogenetic analysis of eIF4E and eIF(iso)4E sequences of different plant species.** The phylogenetic tree was constructed using ClustalW (http://www.ebi.ac.uk/clustalw/). The GenBank accession numbers of the amino acid sequences used are listed in Supplementary Table S2. The two peanut sequences are highlighted in bold and italics.

The eIF4E and eIF(iso)4E protein sequences of other plant species were aligned for phylogenetic reconstruction. In the phylogenetic tree, eIF4E and eIF(iso)4E formed two distinct branches (**Figure 1**). Furthermore, peaeIF4E was clustered with the eIF4E subgroup, whereas peaeIF(iso)4E was classified into the eIF(iso)4E subgroup. Analysis using the conserved domain search service of NCBI confirmed that the two proteins contained the eIF4E family conserved sequence.

### Expression Profiles of *PeaeIF4E*/*PeaeIF(iso)4E* in Different Tissues of Peanut

**FIGURE 2:**
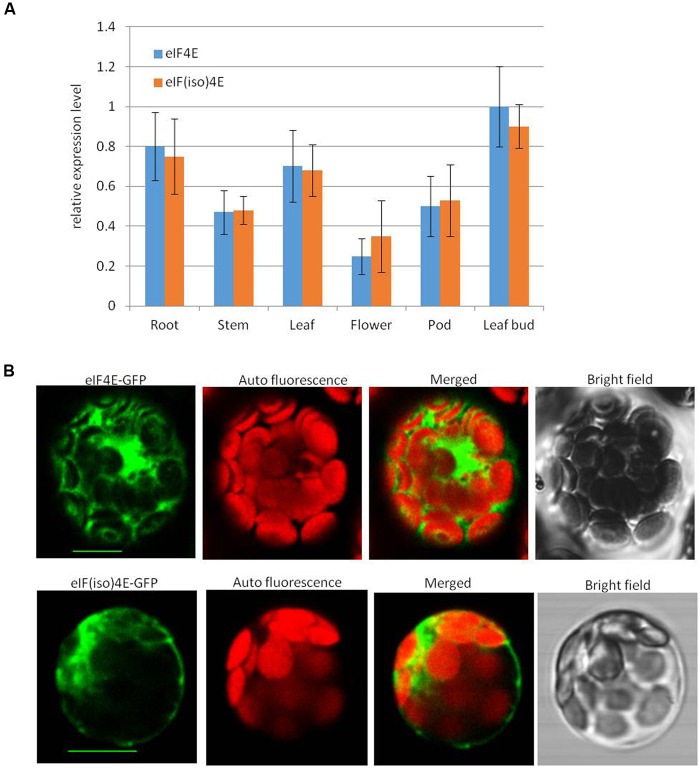
**mRNA transcript levels** **(A)** of PeaeIF4E and PeaeIF(iso)4E and their subcellular localization **(B)**. Relative mRNA expression levels of *peaeIF4E* and *peaeIF(iso)4E* were determined by real-time reverse transcript PCR (RT-PCR). The values represent means of three biological repeats and the value of each biological repeat is the mean of three technical repeats. All values were normalized to the reference gene peanut actin. PeaeIF4E and PeaeIF(iso)4E were fused with green fluorescent protein (GFP) are delivered into protoplasts of *Arabidopsis*. The GFP fluorescence was observed 12–16 h after transfection. Scale bars = 10 μm.

To detect the expression levels of peanut *PeaeIF4E* and *PeaeIF(iso)4E* in various tissues, quantitative real-time PCR was performed. RNA was isolated from various tissues such as the roots, stems, leaves, flower buds, and leaf buds of “Huayu 20.” The expression of the peanut *actin* gene is constant under different conditions and in different tissues (Chi et al., 2012), and was thus selected as a reference. The mRNA transcript levels showed significant differences in different tissues from peanut plants. The expression patterns and the mRNA transcript levels of *PeaeIF4E* and *PeaeIF(iso)4E* were similar in all tissues (**Figure 2A**). The highest transcript levels, for both genes, were observed in the leaf bud and the lowest in flowers (**Figure 2A**).

### Subcellular Localization of PeaeIF4E and PeaeIF(iso)4E in *Arabidopsis*

To test the subcellular localization of PeaeIF4E and PeaeIF(iso)4E, PeaeIF4E and PeaeIF(iso)4E were fused to the GFP by cloning of the ORFs of PeaeIF4E and PeaeIF(iso)4E into the entry vector pGWCm. Recombinant plasmids that expressed the PeaeIF4E-GFP and PeaeIF(iso)4E-GFP fusion proteins were introduced into *Arabidopsis* protoplasts cell. The *Arabidopsis* protoplasts cell were cultured in the dark at 23°C for about 12–16 h and observed by the confocal laser scanning microscopy (Leica SP5). The results suggested that the PeaeIF(iso)4E and PeaeIF4E fusion proteins were present in both the nucleus and the cytoplasm (**Figure 2B**).

### Subcellular Localization of VPg and HC-Pro in *Arabidopsis*

**FIGURE 3:**
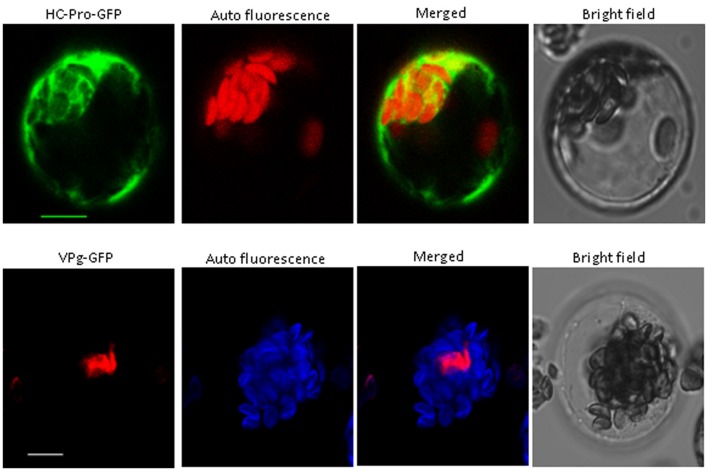
**Subcellular localization of PStV HC-Pro and PStV VPg.** PStV HC-Pro and PStV VPg fused with GFP were transfected into protoplasts of *Arabidopsis*. The GFP fluorescence was observed 12–16 h after transfection. Scale bars = 10 μm.

We obtained the VPg and HC-Pro protein cDNAs from PStV by RT-PCR, and their ORFs were fused to GFP as above. The fusion proteins were expressed in *Arabidopsis* protoplasts as above. The results suggested that the VPg fusion protein was present in the nucleus, and HC-Pro fusion protein was observed in the cytoplasm (**Figure 3**).

### Interaction Analysis between eIF4E/eIF(iso)4E and PStV HC-Pro/PStV Vpg

**FIGURE 4:**
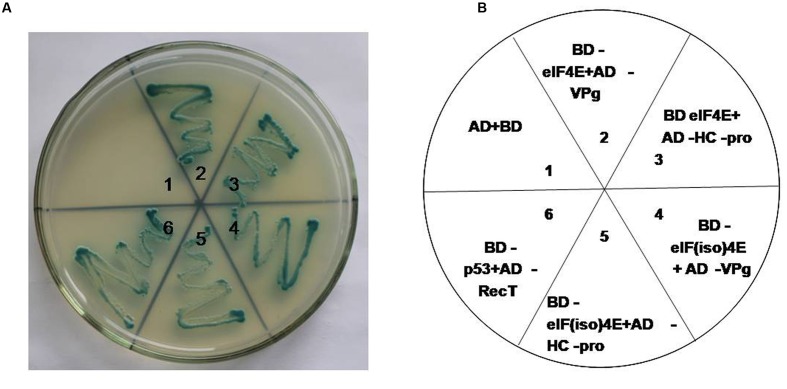
**Yeast two-hybrid assay of protein–protein interaction between the PeaeIF4E/PeaeIF(iso)4E from peanut and PStV-HC-pro/PStV-VPg.** Yeast co-transformants were grown on selective medium SD/-Leu-Trp-His-Ade plus X-α-Gal and incubated for 4 days at 30°C **(A)**. Frame **(B)** corresponds to the clones left **(A)**.

**FIGURE 5:**
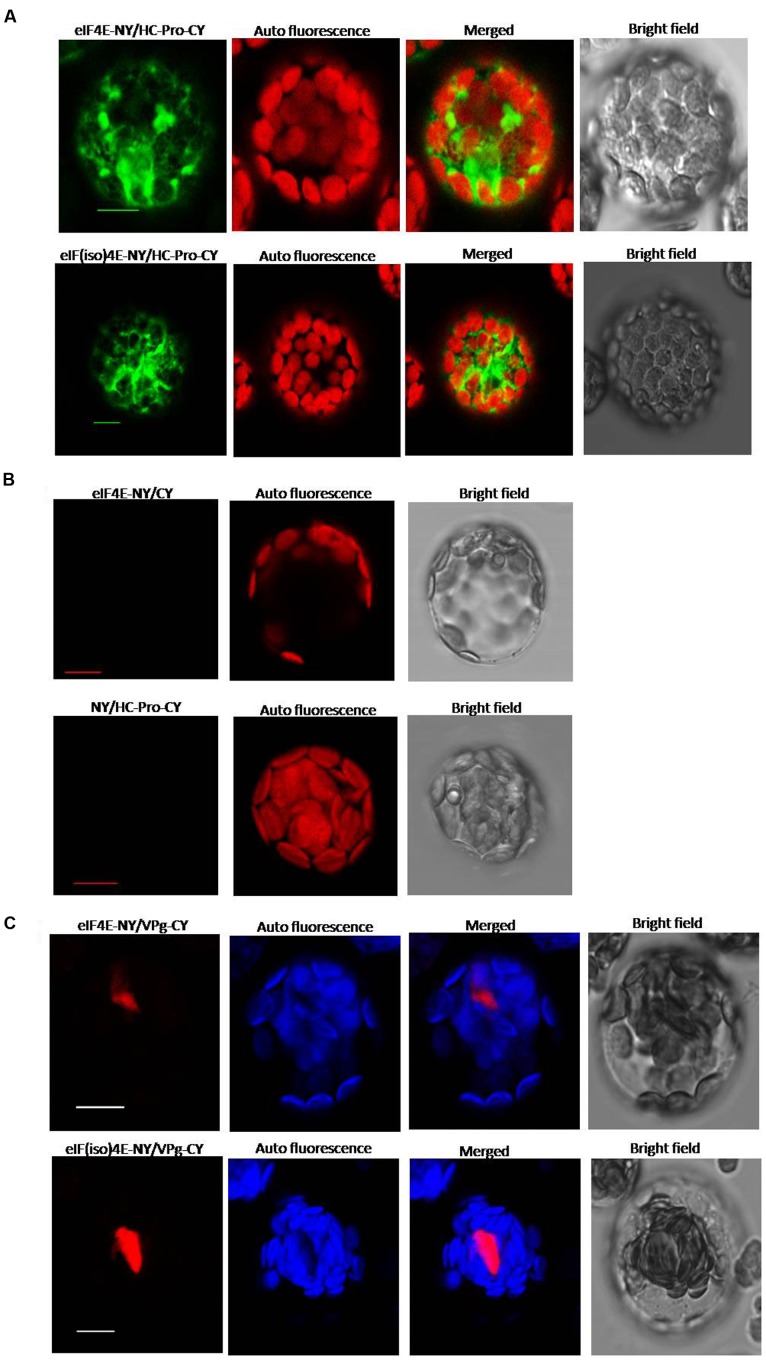
**Bimolecular fluorescence complementation (BiFC) assay showing interaction between PeaeIF4E/PeaeIF(iso)4E and PStV HC-Pro/PStV VPg.** The full-length open reading frame (ORF) of PeaeIF4E/PeaeIF(iso)4E was cloned into the vector pEarleyGate202-NYFP [eIF4E-NY, eIF(iso)4E-NY] and that of PStV HC-Pro/PStV VPg into pEarleyGate202-CYFP (HC-Pro-CY, VPg-CY). The recombinant plasmids were transfected into protoplasts of *Arabidopsis*. Fluorescence was observed at 14–16 h post-transfection by confocal laser-scanning microscopy. Scale bars = 10 μm. **(A)** BiFC analysis of PeaeIF4E/PeaeIF(iso)4E and PStV HC-Pro. **(B)** A range of negative controls. **(C)** BiFC analysis of PeaeIF4E/PeaeIF(iso)4E and PStV VPg.

Yeast two-hybrid analysis was used to determine whether there was an interaction between viral proteins and peanut proteins. Yeast two-hybridization showed interactions between VPg and PeaeIF4E/PeaeIF(iso)4E, and between HC-Pro and PeaeIF4E/PeaeIF(iso)4E (**Figure 4**). The interactions were further confirmed by using BiFC. In this system, the YFP was split into N-terminal and C-terminal fragments, and the PeaeIF4E and PeaeIF(iso)4E were attached to the N-terminal fragment of YFP(eIF4E-NY and eIF(iso)4E-NY). VPg and HC-pro were fused to the C-terminal fragment of YFP(VPg-CY and HC-pro-CY). The eIF(iso)4E-NY+VPg-CY, eIF4E-NY+VPg-CY, eIF4E-NY+HC-pro-CY, and eIF(iso)4E-NY+HC-pro-CY plasmids were then transformed into *Arabidopsis* protoplasts cells. A nuclear fluorescence signal was, respectively, observed in eIF(iso)4E-NY+VPg-CY and eIF4E-NY+VPg-CY combination and the signal was observed throughout the nucleus(**Figure 5C**). Cytoplasmic fluorescence signals were observed in the eIF4E-NY+HC-pro-CY and eIF(iso)4E-NY+HC-pro-CY combinations, and the signals were observed throughout the cytoplasm (**Figure 5A**). As expected, the negative controls, i.e., the combinations of eIF4E-NY+CY and NY+HC-Pro-CY did not emit fluorescence signals (**Figure 5B**). Taken together, these results show that PeaeIF4E/PeaeIF(iso)4E interacts with VPg in the nucleus, whereas PeaeIF4E/PeaeIF(iso)4E interacts with HC-Pro in the cytoplasm.

### Silencing of *PeaeIF4E* and *PeaeIF(iso)4E* Genes Confers Resistance against PStV in Peanut

**FIGURE 6:**
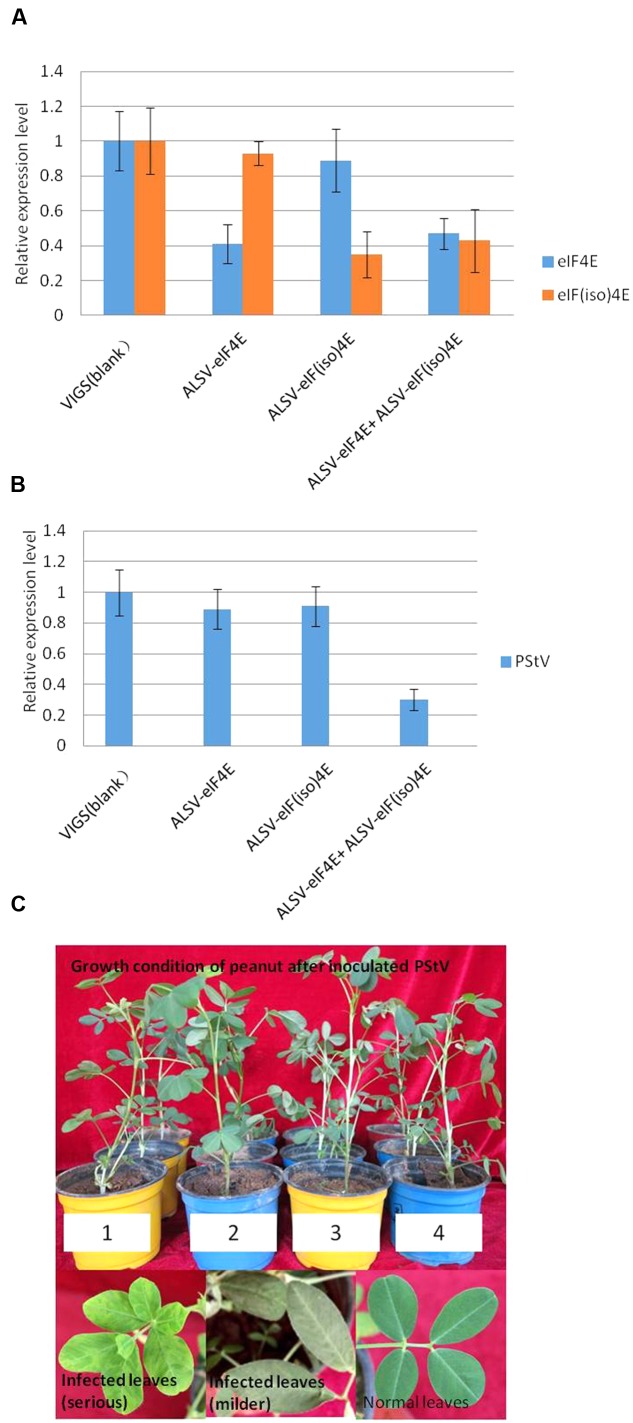
**Real-time PCR analysis for target gene expression in peanut** **(A)**, accumulation of PStV RNA **(B)** and the growth condition and symptoms of peanut after inoculated PStV **(C)**. Virus-induced gene silencing of *PeaeIF4E/PeaeIF(iso)4E* in representative plants belonging to each of the four different treatments. For qRT-PCR detection of the expression of *PeaeIF4E/PeaeIF(iso)4E*, three plants from each group were pooled as one sample and the experiments were performed in triplicate **(A)**. Effects of silencing of *PeaeIF4E/PeaeIF(iso)4E* on PStV infection. The accumulation of PStV RNA in inoculated peanut plants was detected by RT-PCR 15 days after PStV inoculation **(B)**. The growth condition and symptoms of peanut after inoculated PStV **(C)**. Four different treatments peanuts were mechanically infected by PStV. Three plants from each group were pooled as one sample and the experiments were performed in triplicate, 10–14 days later, PStV disease symptom began to appeared. The peanut growth condition after inoculated with PStV, except the different symptoms on peanut leaves, different treatments peanuts growth condition showed no significant differences compared with control. 1, control group; 2, silencing *PeaeIF4E*; 3, silencing *PeaeIF(iso)4E*; 4, silencing both *PeaeIF4E* and *PeaeIF(iso)4E.*

To confirm the role of the *PeaeIF4E* and *PeaeIF(iso)4E* genes in PStV infection, gene silencing was performed. The *PeaeIF4E* (355-nt) and *PeaeIF(iso)4E* (326-nt) fragments were inserted into the ALSV-RNA2 vector. The recombinant viruses (ALSV-eIF4E and ALSV-eIF(iso)4E) were then inoculated into a peanut. Two weeks after inoculation, real-time RT-PCR analysis was performed, which demonstrated that the expression levels of *PeaeIF4E* and *PeaeIF(iso)4E* were significantly lower in the inoculated plants as compared with control (**Figure 6A**), although no significant phenotypic alterations were observed in the transgenic plants (**Figure 6C**). The expression level of *PeaeIF4E* decreased by 60% (upon inoculation with ALSV-eIF4E) while that of *PeaeIF(iso)4E* decreased by 65% (ALSV-eIF(iso)4E inoculation) as compared with control. When inoculated with ALSV-eIF4E+ALSV-eIF(iso)4E, the expression levels of *PeaeIF4E* and *PeaeIF(iso)4E* decreased by 53 and 57%, respectively, as compared with control (**Figure 6A**). Peanut plants where either the *PeaeIF4E* or *PeaeIF(iso)4E* were silenced, showed mosaic symptoms of infection at about 10–14 days after inoculation with PStV. On the other hand, silencing of both *PeaeIF4E* or *PeaeIF(iso)4E* caused the symptoms to appear later at about 18–20 days after inoculation with PStV, and the symptoms were milder as compared with plants with only one gene silenced and the control (**Figure 6C**). Real-time RT-PCR analysis indicated that silencing both *PeaeIF4E* or *PeaeIF(iso)4E* reduced PStV accumulation by 70% compared to control plants. No significant differences were observed between plants in which either *PeaeIF4E* or *PeaeIF(iso)4E* was silenced as compared with controls (**Figure 6B**) suggesting that the two isoforms play overlapping or redundant roles in the virus multiplication cycle.

## Discussion

The cap-binding protein eIF4E/eIF(iso)4E confers resistance to some RNA viruses in specific plant species (Nicaise et al., 2003; Nieto et al., 2006; Ruffel et al., 2006; Hwang et al., 2009). In the present study, we cloned the peanut *eIF4E/eIF(iso)4E* genes and analyzed their protein sequences. Phylogenetic analyses of these sequences demonstrated that *PeaeIF4E* and *PeaeIF(iso)4E* showed high homologies with orthologs from related plant species. *PeaeIF4E* and *PeaeIF(iso)4E* were closely related to their homologs from soybean (*G. max*) and kidney bean (*Phaseolus vulgaris*). The expression levels of *PeaeIF4E* and *PeaeIF(iso)4E* were similar in different peanut tissues with both genes being upregulated in leaf buds and roots and downregulated in flowers (**Figure 2**). Previous studies have also shown that these two genes are upregulated in young tissues and downregulated in mature tissues of *Arabidopsis* and plum, which corroborated our results (Rodriguez et al., 1998; Wang et al., 2013).

Confocal microscopy showed that PeaeIF4E and PeaeIF(iso)4E were both localized in the nucleus and the cytoplasm of *Arabidopsis* cells (**Figure 2B**). In *Chrysanthemum morifolium*, eIF(iso)4E was also localized in the nucleus, cytoplasm, and cytomembrane (Song et al., 2013). In *Arabidopsis*, in quiescent cells, eIF4E was localized in the nucleus, whereas in proliferating cells, this was detected in the cytoplasm. Both in quiescent and proliferating cells, eIF(iso)4E has been observed in the cytoplasm and nucleus (Bush et al., 2009). In mature *Arabidopsis* cells, PeaeIF4E and PeaeIF(iso)4E were localized to both the nucleus and the cytoplasm, but whether the two proteins have different cellular locations during different growth stages needs further investigation. In animals, eIF4E has been detected in both cytoplasm and nucleus, and about 68% of the eIF4E was detected in mammalian nuclei. eIF4E plays different roles depending on its subcellular location; when it is localized in cytoplasm, it functions in translation initiation. When it is localized in the nucleus, it participates in the export of mRNAs that contain 4E-sensitive elements (SE; Iborra et al., 2001; Culjkovic et al., 2007, 2008). The two peanut proteins that localized in different places may play different roles that require further investigation.

Confocal microscopy showed that the VPg fusion protein was localized to the nucleus, whereas the HC-Pro fusion protein was observed in the cytoplasm. In the case of MDMV (*Maize dwarf mosaic virus*) and TuMV (*Turnip mosaic virus*), HC-Pro was detected in the cytoplasm (Li et al., 2001; Zheng, 2011). In the case of *Potato virus Y*, HC-Pro was localized throughout the cytoplasm, whereas it displays different subcellular localization patterns depending on the cellular environment (del Toro et al., 2014). HC-Pro was also distributed throughout the cytoplasm in CABMV (*Cowpea aphid-borne mosaic virus*) infected plants (Mlotshwa et al., 2002). In WYMV (*Wheat mosaic virus*) infected plants, VPg occurred in two forms in the nucleus; one gathered into one or several irregular shape inclusions, whereas the other was evenly distributed across the entire nucleus (Bian, 2013). These results were consistent with the findings of our study as well as with the results of BiFC analysis of HC-Pro and PeaeIF4E/PeaeIF(iso)4E, and that of VPg and PeaeIF4E/PeaeIF(iso)4E. We also observed interactions between VPg and PeaeIF4E/PeaeIF(iso)4E and between HC-Pro and PeaeIF4E/PeaeIF(iso)4E. The interaction between VPg and PeaeIF4E/PeaeIF(iso)4E interactions was observed in the nucleus. These results coincided with the findings on viral protein location in our study. The interaction between VPg and PeaeIF4E/PeaeIF(iso)4E in the nucleus provides additional evidence that both proteins are localized in the nucleus. In potyvirus, the interaction between VPg and eIF4E/eIF(iso)4E plays a major role in cellular transport and localization of RNA (Lellis et al., 2002). HC-Pro and PeaeIF4E/PeaeIF(iso)4E interactions were detected in the cytoplasm but not in the nucleus, which supported the absence of HC-Pro in the nuclei of infected plant cells (Rajamaki and Valkonen, 2003).

The interaction between translation initiation factors and viral proteins is essential for viral replication and infection (Dreher and Miller, 2006; Robaglia and Caranta, 2006). The interaction between eIF4E/eIF(iso)4E and HC-Pro/VPg may be necessary for potyvirus infection and amplification. The silencing of both *PeaeIF4E* and *PeaeIF(iso)4E* conferred moderate resistance against PStV in peanut, as evidenced by symptom delay and reduced virus accumulation. This findings strongly suggest that PeaeIF4E and PeaeIF(iso)4E play important roles to facilitate virus infection and that they are functionally interchangeable. The silencing of *PeaeIF4E* and *PeaeIF(iso)4E* hindered the interaction between the host and the virus, which in turn prevented infection and viral replication in the host. Furthermore, viral accumulation was lower in gene-silenced peanut plants. Silenced plants with decreased expression of PeaeIF4E and/or PeaeIF(iso)4E did not phenotypically differ from control plants. In tobacco, antisense depletion of either eIF4E and two eIF(iso)4E isoforms displayed normal development, but antisense depletion of both eIF4E and eIF(iso)4E resulted in semi-dwarf phenotype (Combe et al., 2005). It is possible that the remaining low levels of expression of PeaeIF4E and PeaeIF(iso)4E in the silenced plants were sufficient to sustain peanut growth. Alternatively, it is possible that the two genes are dispensable for peanut growth and that some other genes possess complementary functions.

In other virus-host compositions, simultaneous mutations in the eIF4E and eIF(iso)4E genes result in a decrease in viral resistance, such as resistance to *Pepper veinal mottle virus* (PVMV) and ChiVMV in pepper (Ruffel et al., 2006; Hwang et al., 2009). Furthermore, knocking down the *eIF(iso)4E* in peach plants results in peach resistance to PPV (Cui and Wang, 2016). Silencing of the *eIF(iso)4E* gene in plum confers resistance to PPV (Wang et al., 2013) and silencing the *eIF4E* gene in melon plants confers upon it broad-spectrum viral resistance (Rodriguez-Hernandez et al., 2012). These reports suggest that these viruses probably utilize one or two translation initiation factors during infection. PStV may use either translation initiation factors of *PeaeIF4E* and *PeaeIF(iso)4E* during its infection because silencing only one gene does not confer resistance to PStV in peanut. Our study suggests that the eIF4E/eIF(iso)4E gene may be utilized in increasing PStV resistance in peanut by gene silencing, gene mutation, or the TILLING strategy (Ruffel et al., 2002; Kanyuka et al., 2005; Stein et al., 2005; Piron et al., 2010). We have a variety of peanut cultivars that could be employed in TILLING to detect allelic variants of a target gene.

Therefore, the findings of the present study suggest that eIF4E/eIF(iso)4E plays important roles in the PStV infection cycle and may serve as a novel method for increasing the PStV resistance in economically important peanut cultivars. The two genes may also be used as genetic resources for improving PStV resistance in peanut breeding programs.

## Author Contributions

MX, HX, JW, and JY: Design of the work, analysis, and interpretation of data. LX and YC: Final approval of the version to be published and agreement to be accountable for all aspects of the work.

## Conflict of Interest Statement

The authors declare that the research was conducted in the absence of any commercial or financial relationships that could be construed as a potential conflict of interest.
