# Supplementary material for: Translation Initiation Factor eIF4E and eIFiso4E Are Both Required for Peanut stripe virus Infection in Peanut (Arachis hypogaea L.)
Source: Front Microbiol. 2017 Mar 10;8:338. doi: 10.3389/fmicb.2017.00338 (PMC5344889; doi:10.3389/fmicb.2017.00338)
Supplement: Supplementary file 1 [file Table_1.doc]

Table S1. Sequences of primers used in this study.

| Primer name | Primer sequence (5**´**-3**´**) |
| --- | --- |
| 4E-F | ATAACATTCAYCAYCCKRGY |
| 4E-R | ACTCCTTCCACTGYTTTCCA |
| eIF4E2-F | CGAGAGAGAGCAAAAATGGTT |
| eIF4E2-R | CCGCACACAAATCTGGAAATA |
| R3'CDS | AAGCAGTGGTATCAACGCAGAGTACTTTTTTTTTTTTTTTTTTTTTTTTTTTTTTVN |
| RUP-L | CTAATACGACTCACTATAGGGCAAGCAGTGGTATCAACGCAGAGT |
| RUP-S | CTAATACGACTCACTATAGGGC |
| RUNP | AAGCAGTGGTAACAACGCAGAGT |
| GSP1 | AGCACAAGATTGAGCCTAAATGGGAG |
| GSP2 | TGGTGATGAGATTTGCGGAGC |
| R5'AD | AAGCAGTGGTATCAACGCAGAGTACGCGGG |
| R5'PCR | AAGCAGTGGTATCAACGCAGAGT |
| I3'CDS | GCTGTCAACGATACGCTACGTAACGGCATGACAGTGTTTTTTTTTTTTTTTTTT |
| IUP-W | GCTGTCAACGATACGCTACGTAACG |
| IUP-N | CGCTACGTAACGGCATGACAGTG |
| IE4PJ3'-F | TTGGAAAACAGTGGAAGGAG |
| IE4PJ3'-R | GCACACAAATCTGGAAATAC |
| IE4PJ5'-F(qc) | AGAAAGAAACCGAGAGAGAGC |
| IE4PJ5-R | TAGGGTCCTCCCATTTAGG |
| IE4qc-R | ATCCGCACACAAATCTGG |
| 4E(iso)1-F | TACAACAAACCCTCATCG |
| 4E(iso)1-R | ATAACAGCCTAGCAGCAC |
| ORF4E-F | ATGGTGGTTGAAGATAC |
| ORF4E-R | CCTATGACGTATTTGTT |
| 4E(isoORF)F | ATGGCAACCGAAACAG |
| 4E(isoORF)R | CCTACACTGTACCGACT |
| YG4E-F | AACCCTAACAACGAAAACG |
| YG4E-R | CGAACCAGAAGGTCCAAGA |
| YG4IE-F | ATGATGGCTTTGATTGGG |
| YG4IE-R | GTTTGCTGCGGTCTTTGT |
| actin-F | TTGGAATGGGTCAGAAGGATGC |
| actin-R | AGTGGTGCCTCAGTAAGAAGC |
| GB-4E-F | CATATGATGGTGGTTGAAGATA (*Nde*Ⅰ) |
| GB-4E-R | CTGCAGTATGACGTATTTGTTT (*Pst*Ⅰ) |
| GB-4IE-F | GAATTCATGGCAACCGAAACA (*EcoR*Ⅰ) |
| GB-4IE-R | CTGCAGTACACTGTACCGACTCTT (*Pst*Ⅰ) |
| GA-HC-Pro-F | GAATTCTCTCAAATTCCTGAAAT (*EcoR*Ⅰ) |
| GA-HC-Pro-R | CTCGAGTCCAACTCTGTAATACTT  (*Xho*Ⅰ) |
| GA-Vpg-F | CATATGGGAAAGAAGCGCATGATA (*Nde*Ⅰ) |
| GA-Vpg-R | CTCGAGTTCAACAGCGACCTCTT (*Xho*Ⅰ) |
| 4E-F | ATGGTGGTTGAAGATAC |
| 4E-R | CCTATGACGTATTTGTT |
| 4E(iso)-F | ATGGCAACCGAAACAG |
| 4E(iso)-R | CCTACACTGTACCGACT |
| PV-F | ATGGGAAAGAAGCGCA |
| PV-R | CCTTCAACAGCGACYTC |
| PH-F | ATGTCTCAAATTCCTGAA |
| PH-R | CCTCCRACTCTGTAATACTT |
| 4E(CM)-F | CCGCTCGAGAACTCTTGGACCTTCTG (*Xho*Ⅰ) |
| 4E(CM)-R | CGCGGATCCCCTGCCTATTCCTGAC (*BamH*Ⅰ) |
| 4E(isoCM)-F | CCGCTCGAGAAGACTGGGATTGAGC (*Xho*Ⅰ) |
| 4E(isoCM)-R | CGCGGATCCTTTGATGATCGGGTTC (*BamH*Ⅰ) |
| YGPStV-F | ATTGTTCCTCGGCTTCAG |
| YGPStV-R | TCAGTTTGCTCTGGCTTGT |
| YG4E-F | AACCCTAACAACGAAAACG |
| YG4E-R | CGAACCAGAAGGTCCAAGA |
| YG4IE-F | ATGATGGCTTTGATTGGG |
| YG4IE-R | GTTTGCTGCGGTCTTTGT |
| actin-F | TTGGAATGGGTCAGAAGGATGC |
| actin-R | AGTGGTGCCTCAGTAAGAAGC |

Note: underlined sequences indicate restriction enzyme sites, the names of which are shown in brackets.
