# Supplementary material for: Translation Initiation Factor eIF4E and eIFiso4E Are Both Required for Peanut stripe virus Infection in Peanut (Arachis hypogaea L.)
Source: Front Microbiol. 2017 Mar 10;8:338. doi: 10.3389/fmicb.2017.00338 (PMC5344889; doi:10.3389/fmicb.2017.00338)
Supplement: Supplementary file 2 [file Table_2.docx]

Table S2. Names, Plant Species and GenBank accession numbers of eIF4E and eIF(iso)4E used for Fig.1

| Names | Species | GenBank accession numbers accession numbers |
| --- | --- | --- |
| CaeIF4E | *Cicer arietinum* | XP_004485968 |
| CpeIF4E | *Carica papaya* | ACN38307 |
| GmeIF4E1 | *Glycine max* | NP 001237528 |
| GmeIF4E2 | *Glycine max* | XP_003546060 |
| MteIF4E | *Medicago truncatula* | XP_003593833 |
| NteIF4E | *Nicotiana tabacum* | CBJ34332 |
| PdeIF4E | *Prunus domestica* | AGE81987 |
| **PeaeIF4E** | *Arachis hypogaea L.* | HE985069 |
| PseIF4E1 | *Pisum sativum* | ABG35116 |
| PseIF4E2 | *Pisum sativum* | ADK97766 |
| PveIF4E1 | *Phaseolus vulgaris* | ACQ59192 |
| PveIF4E2 | *Phaseolus vulgaris* | AGI41289.1 |
| SleIF4E | *Solanum lycopersicum* | XP 004231545 |
| VveIF4E | *Vitis vinifera* | XP_002267488 |
| CaeIF(iso)4E | *Cicer arietinum* | XP_004497820 |
| CmeIF(iso)4E | *Cucumis melo* | ABY56090 |
| GmeIF(iso)4E | *Glycine max* | XP_003535996 |
| LseIF(iso)4E | *Lactuca sativa* | AAP86603 |
| MteIF(iso)4E XP_003590157)XP_003590157) | *Medicago truncatula* | XP_003590157 |
| PdeIF(iso)4E  () | *Prunus domestica* | AGE81988 |
| **PeaeIF(iso)4E** | *Arachis hypogaea L.* | KF956378 |
| PseIF(iso)4E1 | *Pisum sativum* | ABH09880.1 |
| PseIF(iso)4E2 | *Pisum sativum* | BAM28884.1 |
| PveIF(iso)4E | *Phaseolus vulgaris* | ABU54805 |
| RceIF(iso)4E | *Ricinus communis* | XP 002528368 |
| SteIF(iso)4E | *Solanum tuberosum* | CBJ34336 |
| TceIF(iso)4E | *Theobroma cacao* | EOY06160.1 |
| VveIF(iso)4E | *Vitis vinifera* | XP_002285444 |

Note: the two peanut sequences are highlighted in bold.
